# The issue of “combined homicide”: a review of the literature and an explanatory case including blunt force trauma, strangulation and stab wounds

**DOI:** 10.1007/s12024-025-01007-6

**Published:** 2025-04-16

**Authors:** Alberto Amadasi, Marc Windgassen

**Affiliations:** 1 Institute of Legal Medicine and Forensic Sciences, Charité-Universitätsmedizin Berlin, Turmstrasse 21, 10559 Berlin, Germany; 2 Institute of Legal Medicine, University Hospital Jena, Am Klinikum 1, 07747 Jena, Germany

**Keywords:** Combined homicide, Multiple methods, Hammer blunt force, Strangulation, Stab wounds

## Abstract

Among the various methods employed in the commission of homicide, the occurrence of what is termed a “combined homicide” has been documented, where the perpetrator uses more than one method to bring about the victim’s death. This article presents the case of a 59-year-old woman who was murdered by her husband in a premeditated manner, using three distinct methods in succession: blunt force trauma inflicted by hammer blows to the head, asphyxia through strangulation with a cord and sharp force injury resulting from multiple stab wounds to the abdomen. In the literature, only a limited number of cases have been analyzed under such a classification and there is currently no widely accepted nosological definition. This case shares similarities with instances of “complex suicide,” particularly due to the use of multiple injurious methods. However, it does not fall within the “overkill” category, as the intent was not to inflict excessive harm on the body, but to kill the victim. Therefore, it is crucial to establish a clear and consistent definition of “combined homicides” to ensure accurate classification and facilitate a comprehensive forensic investigation.

## Introduction

In the forensic literature, several cases of homicide in which multiple methods or mechanisms are used simultaneously or in combination have been described. These types of homicides are often complex and can involve various forms of violence, such as blunt force trauma, strangulation, stabbing, shooting, poisoning, and others [1–7].

While the term “combined homicide” rarely appears as a distinct category in forensic medicine, several related fields within forensic criminology and pathology address these types of killings [8]. For example, forensic psychology, criminal profiling, crime scene analysis, as well as legal and investigative challenges explore cases involving multiple methods of homicide. These cases exhibit certain similarities with the concept of “complex suicides”, a term that refers to suicides involving multiple distinct methods or mechanisms of self-harm, but differ in intent, as suicides are self-inflicted while homicides are perpetrated by another individual. Unlike more straightforward suicides, complex suicides present a unique challenge to investigators, clinicians, and researchers due to their complex nature. They may involve combinations of lethal methods, such as asphyxiation, poisoning and blunt force trauma, or they may feature a sequence of self-inflicted injuries that complicate the determination of intent and the understanding of underlying motivations. In forensic and psychological literature, complex suicides refer to instances in which an individual employs multiple distinct methods to carry out the suicidal act [9–14]. These cases may be tough to interpret, often confusing investigations, as the combination of methods may resemble homicide or accidental death. However, they are distinguished by characteristics that suggest suicidal intent. Various types of complex suicides have been described in the literature, each involving specific combinations of methods, such as asphyxiation and physical trauma, poisoning and asphyxiation, fire and asphyxiation, gunshot wounds and poisoning, drowning or jumping from a height with self-inflicted wounds. Such cases therefore require a multidisciplinary approach, blending pathology, psychology, and investigative techniques. Due to their forensic complexity, combined homicides also show similarities with the concept of “overkill”. It refers to the use of excessive force or multiple methods in the commission of a homicide, where the degree of violence far exceeds what is necessary to cause death. This phenomenon, often observed in violent crimes, presents a unique challenge to forensic investigators, as it may indicate particular psychological or emotional states, such as intense anger, rage, or sadism. Overkill is typically characterized by repeated or disproportionate injuries to the victim along with completely excessive violence, such as multiple stabbings, blunt force trauma, or several gunshot wounds. In forensic terms, overkill is not only about the sheer physical violence but can also provide valuable insights into the perpetrator’s psychological state [14–16]. The use of excessive violence may indicate a perpetrator’s psychological profile and emotional condition at the time of the crime. Overkill may reflect a premeditated act, but it can also arise from an impulsive outburst of violence driven by extreme psychological distress. This form of excessive violence can serve as a critical clue in differentiating homicides from suicides and accidents, as it typically points to a deliberate and intentional act of aggression. In distinction from overkill a very illustrative case of homicide with a combination of different methods is presented, which offers insights for a comprehensive review of the concept of “combined homicide”.

## Case report

A 59-year-old woman was found dead inside her home after her 72-year-old husband called the police admitting to having killed her. His statement that he was unable to cope with caring for his wife, who had been dependent on support since suffering a stroke, suggests a motive, but the preparation of the weapons and the scene may indicate a level of premeditation. The perpetrator was also affected by mobility impairments as well as advanced chronic obstructive pulmonary disease (COPD) with detectable breath shortness. He had lured her into the former children’s room of their apartment under a pretext, where he had already laid out a hammer, a knife and an electric cable. According to the men´s statement, he hit her several times on the back of the head with the hammer from behind while she was standing and then choked her with the cable while she was lying on the floor. As she could still be heard gasping, he then stabbed her several times to complete unresponsiveness with the knife.

Within the apartment, the following items were found in the room where the body was discovered: a kitchen knife with a plastic handle and a single-edged blade approximately 13 cm in length and 2.5 cm in maximum width; a hammer with a wooden handle and a metal head, with an overall length (handle plus head) of 33 cm, a head width of 3 cm, a thickness of about 2.5 cm on the flattened side, and about 0.4 cm on the pointed side; and an electric vacuum cleaner cable with a length of approximately 2 m and a thickness of approximately 6 mm. All of these items were found to have bloodstains.

Before the autopsy, the body was subjected to a full CT scan with a 3D reconstruction and toxicological analyses were conducted on the victim’s fluids and tissues after the autopsy.

**Fig. 1:**
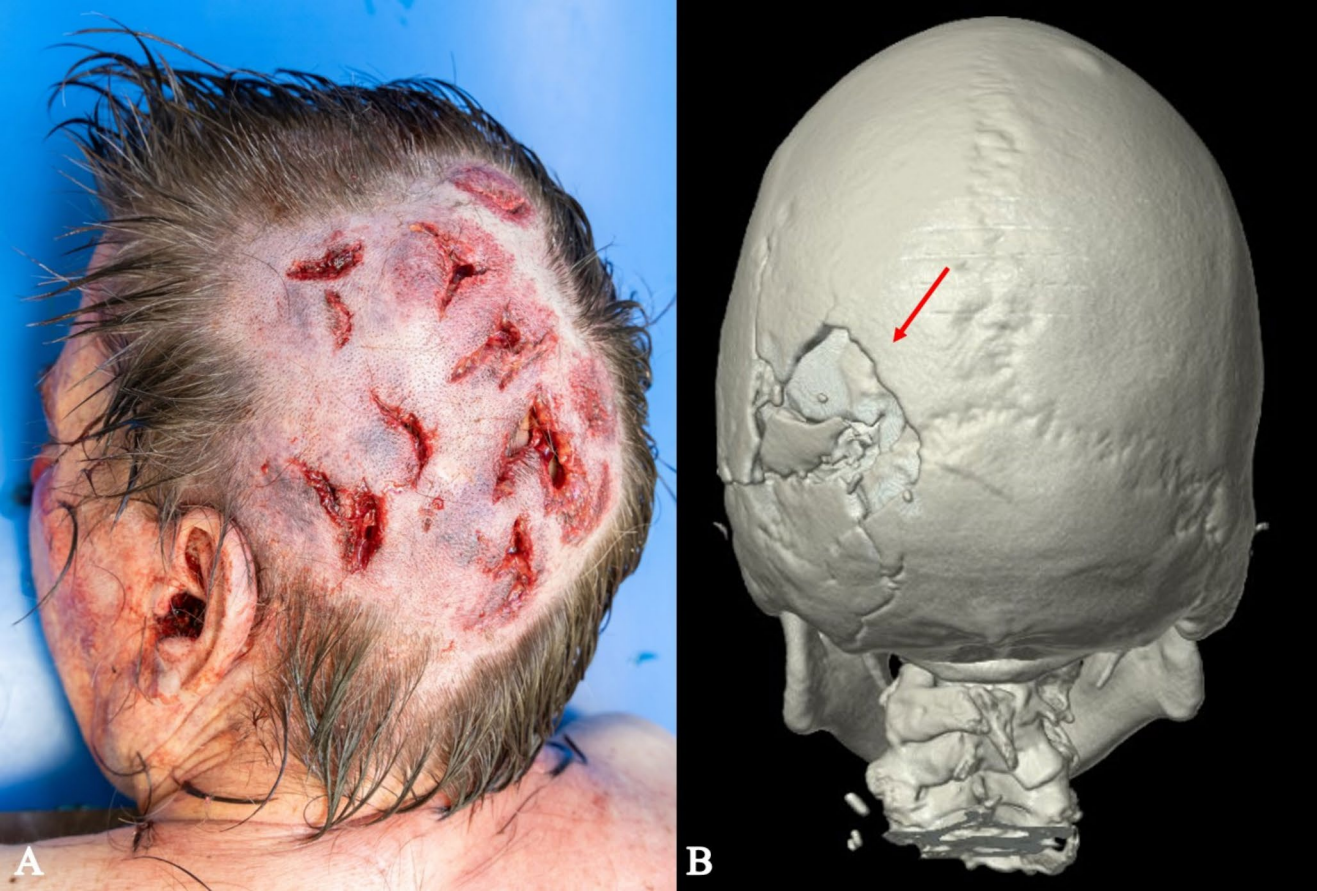
Multiple hammer blunt force injuries on the scalp (**A**) and CT 3d reconstruction of the depressed fracture complex in the right occipital region (red arrow) (**B**)

**Fig. 2:**
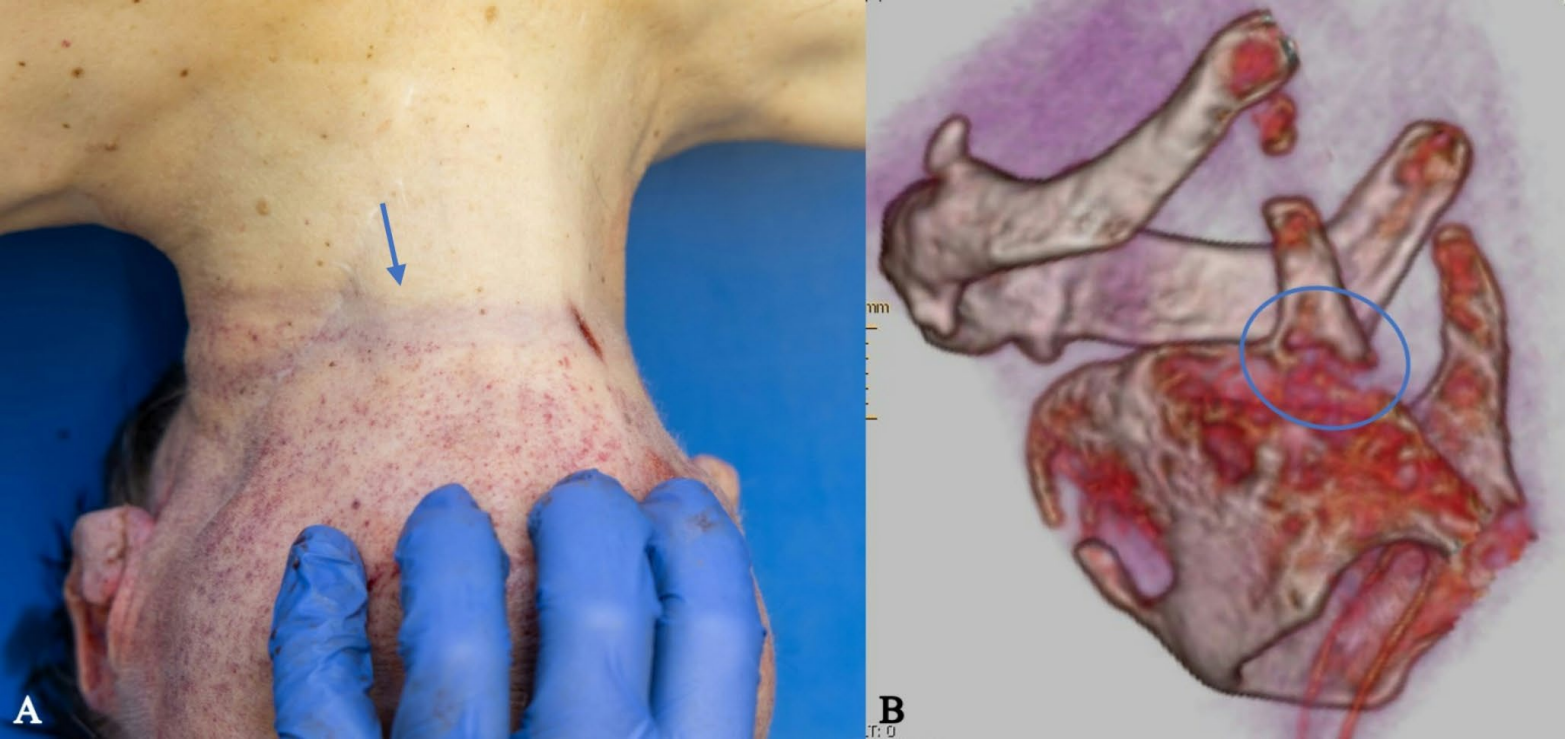
**A**: Linear strangulation sign with an almost horizontal course around the neck (blue arrow) and considerable congestion with diffuse petechiae of the facial skin. **B**: 3D reconstruction of the cartilaginous skeleton of the larynx with fracture of the left upper thyroid horn (blue circle)

**Fig. 3:**
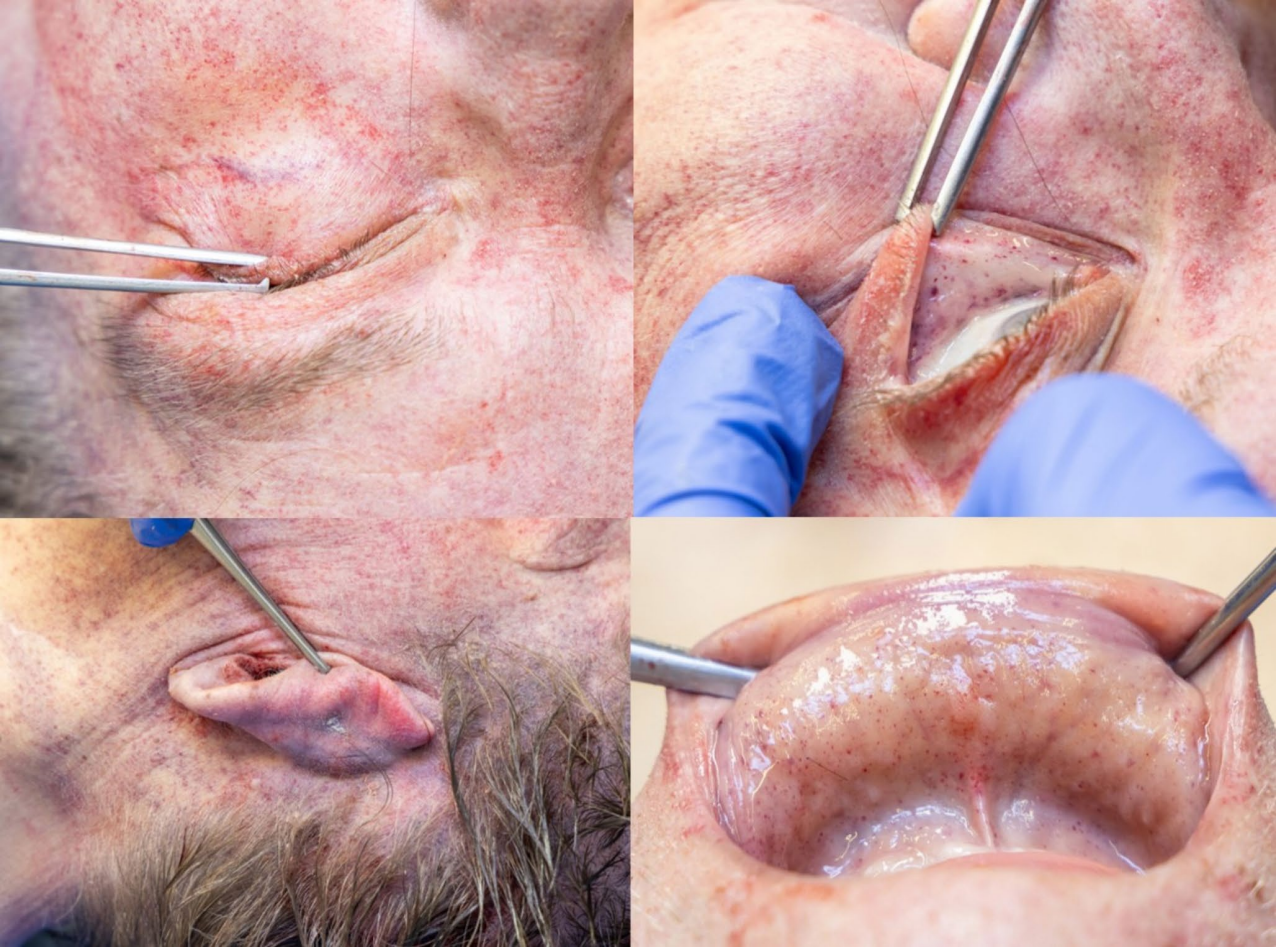
Clockwise: extensive petechiae of the eyelids, conjunctiva, oral vestibular mucosa, retroauricular region

**Fig. 4:**
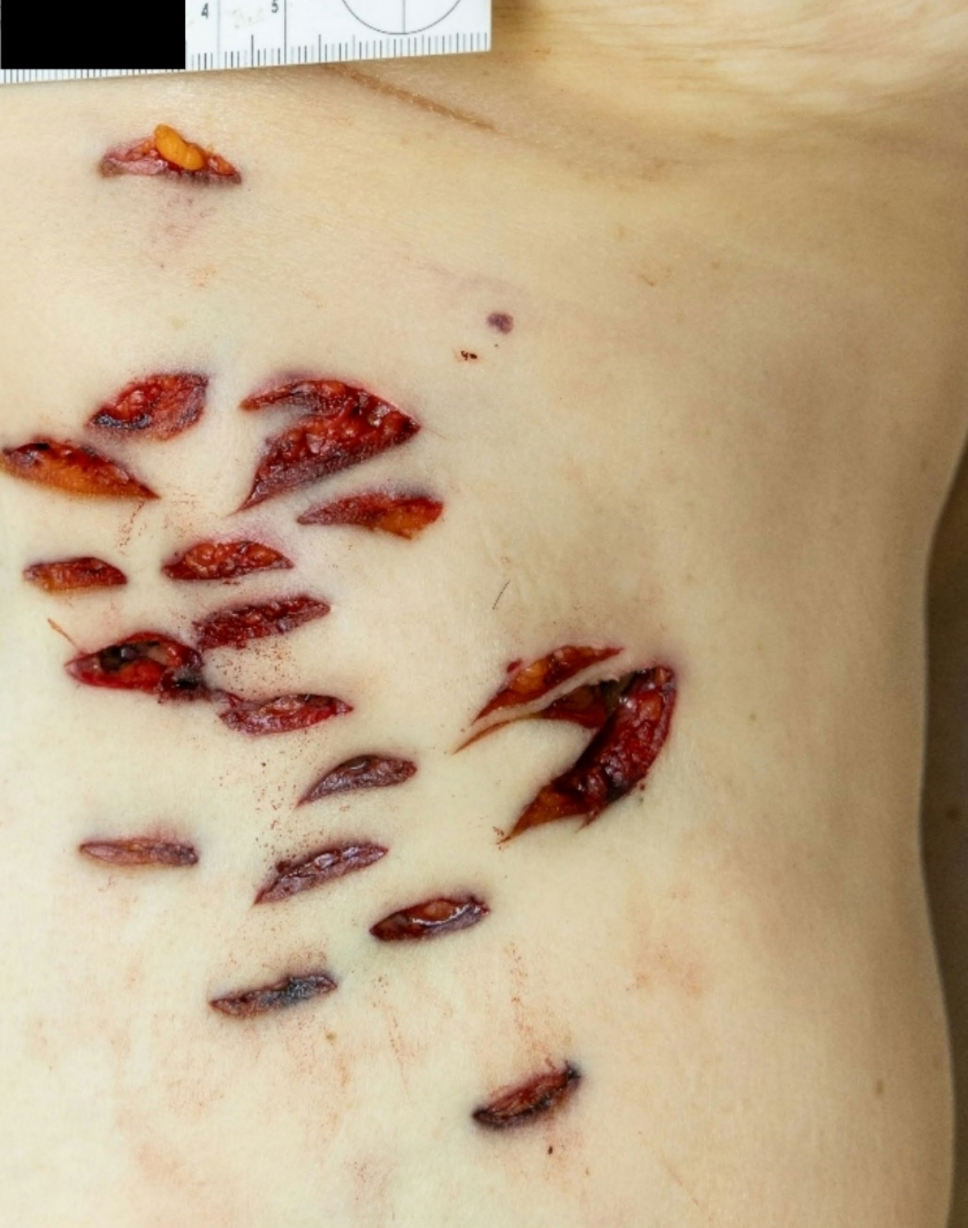
Multiple stab wound in the left upper abdomen

The corpse was 155 cm tall and weighed 51 kg. The following findings were detected:

- *Craniocerebral trauma*: in the parieto-occipital and occipital regions, a total of 11 lacerated wounds were found, with a maximum length of approximately 3 cm. The wounds were partly “arc-shaped”, partly “star-shaped” or “Y-shaped”, with irregular and blood-infiltrated margins, and several tissue bridges within the wounds (Fig. 1A). An imprinted, depressed fracture complex on the left occipital area with extension into the middle and posterior cranial fossa (Fig. 1B). Abundant free air was radiologically detected in the cranial cavity, along with hemorrhages in the trabeculae of the arachnoid and contusion hemorrhages in the left cerebral hemisphere.
- *Signs of strangulation*: Almost horizontal around the neck, slightly imprinted, band-shaped strangulation mark. Rupture of the left upper thyroid horn (Fig. 2A, B). Massive congestion above the jugular plane with innumerable pointed blood spots (petechiae) in the facial skin, the skin of the eyelids, the conjunctiva, the posterior ear region, the oral vestibular mucosa (Fig. 3). Hyperinflation of the lungs.
- *Abdominal stab wounds*: 18 closely anatomically related stab wounds on the left front of the abdomen (Fig. 4) with regular margins and a maximum size of 3 × 1.4 cm, partly merging with each other, with very shallow ascending stab path with penetration of the soft tissue and chest muscles. Corresponding injuries of diaphragm, pericardium, heart, left lung, stomach, liver, upper small intestine along with 50 ml blood in the abdomen and 20 ml blood in the pericardium were detected.

The following pre-existing conditions were detected: older tissue degeneration of the left hemisphere of the brain (as remnants of the previous stroke), adhesions and fibrosis of the lungs as a possible consequence of past inflammatory processes, hemangioma (benign tissue growth) of the liver, benign tissue growths of the uterus (fibroids), cysts on both ovaries. Toxicological analyses detected Mirtazapine in the venous blood. In the stomach content, the active substances salicylamide and acetylsalicylic acid were detected. The concentration of mirtazapine measured in the venous blood of the deceased lied within the therapeutic range. There was no evidence of alcohol intoxication. The findings indicated no relevant impairment of perception, reaction, or motor abilities due to alcohol, medications, or drugs.

The morphology of the injuries causing the massive craniocerebral were detected to be consistent with blows with a hammer [17, 18] and with the resulting depressed fractures typical of the application of such contusive injury to a small surface area [19–21] therefore also from a dimensional perspective with the instrument found at scene. The type and distribution of the injuries could be attributed to violence inflicted by a third party, the blows were inflicted from behind. The injury pattern can be described as fatal in itself; the fact that there was no more serious bleeding inside the skull is due on the one hand to the massive loss of blood to the outside through the head injuries and on the other hand to the strangulation process, which accelerated the onset of death. While both methods were fatal, the sequence of injuries likely played a significant role in the timing of death. This is because the findings of a strangulation were presented as a second significant injury complex [22]. The strangulation mark found would typically be compatible with the cable found on the scene. The massive congestion not only demonstrated the intensity and circulatory relevance of the incident, but also in particular that death had not yet occurred at the time of strangulation. As a third complex, a total of 18 abdominal stab wounds were found. The wound morphology suggested a single-edged tool, the course of the stabbing channels with a very shallow slope suggests that they were inflicted in a lying position. Injuries to various internal organs (lungs, liver, intestines, stomach) and, in particular, a total of five openings of the heart resulted in only scarce haemorrhages in these areas, which suggests that they were inflicted during the agonal phase. However, these injuries itself would have also been fatal. Overall, the pattern of the injuries was consistent with the statements made by the suspect about the sequence of events. There were no defensive injuries, nor would they have been expected in view of the massive blunt force against the head from behind and consecutive unconsciousness. Following the trial, the man was subsequently sentenced to imprisonment for homicide.

### Literature review

The forensic literature on homicides involving the combination of different methods is quite limited, and the terminology used is not consistent. As previously mentioned, this type of homicide should not be considered “overkill” because the techniques employed are intended to cause the death of the victim, and the homicidal actions cease once death occurs, without any post-mortem brutality typically seen in overkill cases.

Some articles describe homicides involving various asphyxiation mechanisms. In 2003, Lupascu et al. [23] reported the case of a 75-year-old woman killed using three different asphyxial methods: smothering, manual strangulation, and traumatic asphyxia due to thoracic compression, referring to this as a “combination” of methods. Similarly, Pramanik (2014) [24] described the case of a 74-year-old man killed through a combination of three asphyxial methods: traumatic asphyxia, ligature strangulation, and smothering with a plastic bag. Pramanik used the terms “multifactorial homicidal asphyxia” and referred to it as a “combination” of methods. In the case described by Das et al. in 2016 [25] three different asphyxial methods were reported, i.e. manual strangulation, smothering and traumatic asphyxia by thoracic compression and referred as a “combination” of asphyxial methods.

Regarding the use of different types of injuring mechanisms belonging to different forensic categories, Choudhary et al. (2017) [5] reported the case of a 15-year-old girl who was murdered through an “association” of two methods: strangulation followed by throat cutting. The authors referred to this as an “association of dual methods”. Faisal et al. (2015) [6], in describing the case of a 30-year-old man killed by gunshot wounds, throat slashing, and stab injuries, referred to it as an “unusual homicide” without providing a specific nosological reference to the coexistence of the different methods of injury. A case study conducted by Behera et al. (2021) [4] examined autopsy cases from New Delhi, reporting a total of 187 cases of “homicide by multiple fatal methods.” The term “combination of methods” was also used in their article. They found that, in cases involving male victims, the most common methods were a combination of head trauma and stabbing to the chest or abdomen, followed by strangulation and smothering. In cases involving female victims, the predominant combination was ligature strangulation and smothering, with the second most common being a combination of smothering and throttling.

In a psychological study conducted by Kamaluddin et al. (2014) [7] among Malaysian male homicide offenders, cases involving ‘multiple methods of killing’ were also examined. The authors found that the use of multiple methods was more frequently observed in “premeditated” murders than in “crimes of passion,” with revenge being a common motive. Instances of such methods included combinations of stabbing and strangulation, or slashing combined with physical force. Various studies have identified the use of multiple homicidal methods in killings. Some studies suggest that females are more often victims of such methods compared to males, potentially due to factors such as gender-based violence or the typically higher vulnerability of females in domestic settings [26, 27], the most commonly observed methods include sharp force trauma, blunt force trauma and strangulation, often used in various combinations [1–8].

The reasons for using multiple methods can vary. One explanation is the perpetrator’s determination to ensure the victim’s death and prevent any chance of survival. Extreme hatred or emotional frenzy may also contribute to excessive violence. Additionally, the involvement of multiple attackers or prolonged resistance from the victim can lead to the use of multiple methods. Another possible factor is the perpetrator’s fear of being caught if the victim survives, calls for help, fights back, or attempts to flee [28]. Strong emotions such as jealousy, hatred, and the desire for revenge are also common motivations for inflicting multiple injuries The availability of weapons and the choice to use them could further influence the decision to employ multiple methods [29].

The definition of “combined homicide” has been discussed in some studies [7, 8, 29, 30] with the term referring to the use of different homicidal methods. These cases involve the use of multiple techniques, either by one or more perpetrators, to achieve the victim’s death. Such murders typically occur when multiple perpetrators use different methods to conceal the true cause of death, or when an initial attack with one weapon fails and is followed by a more effective method. Other factors may include the availability of weapons at the scene, the sudden appearance of potential witnesses, or other unpredictable circumstances.

Studies have shown that the use of multiple methods of killing is often associated with premeditated homicide, in contrast to passion-driven murders. For example, those committing premeditated murder may employ a combination of methods, such as stabbing and strangulation, to ensure that the victim is dead.

### Registered cases of “combined homicide” at the Institute of Legal Medicine and Forensic Sciences of Berlin, Germany

**Fig. 5:**
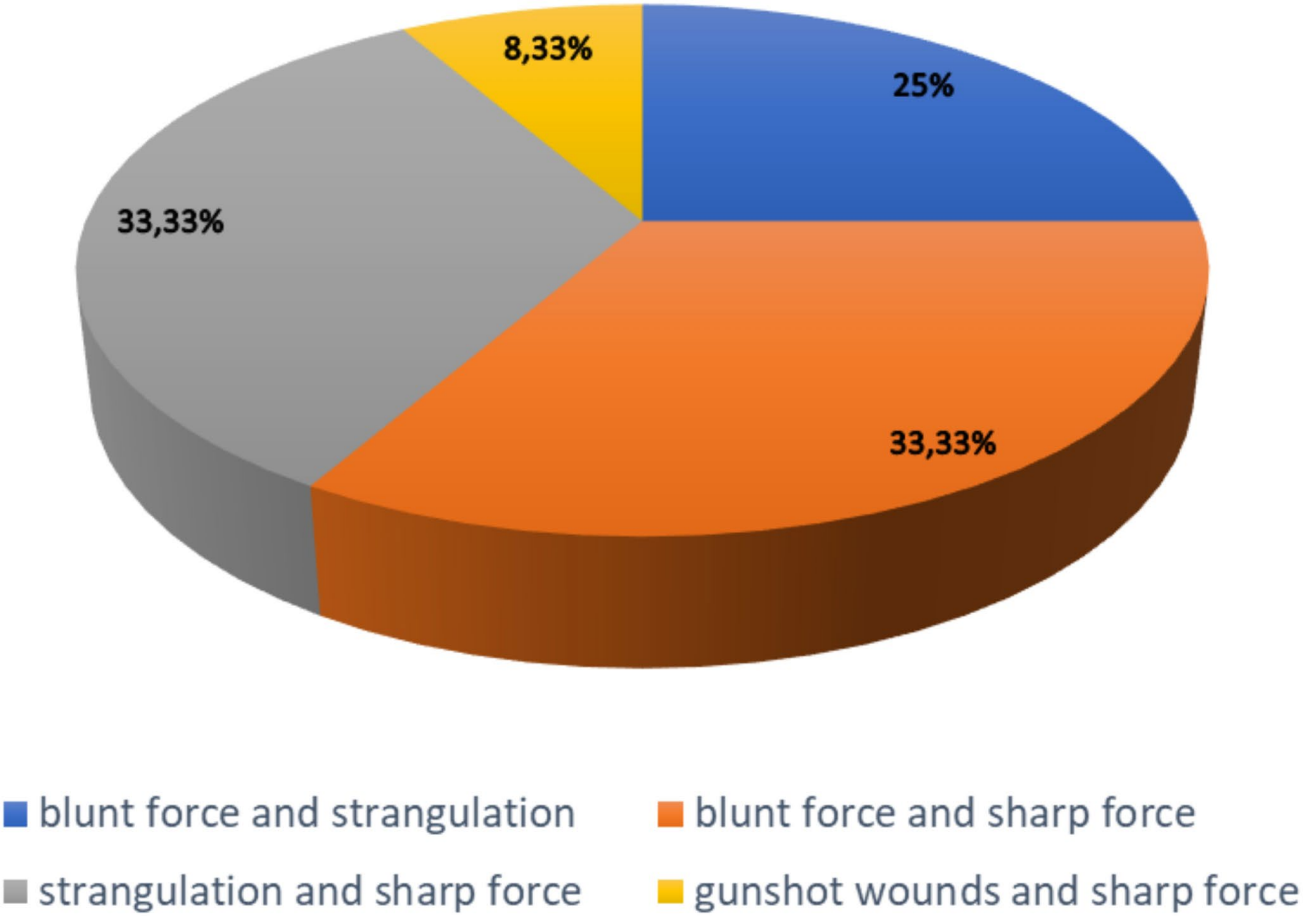
Percentage of “combined homicide” types in Berlin over the period 2010–2023 (total: 12 cases)

In a survey carried out on autopsies in homicide cases at the Institute of Legal Medicine and Forensic Sciences of Berlin, Germany, between 2010 and 2023, out of a total of 433 autopsies for homicide cases, 12 cases of “combined homicide” were detected (2.77%), all due to the combination of two different methods. Among these, 4 cases were due to the combination of blunt and sharp force injuries (33,33%), 4 to strangulation and sharp force injuries (33,33%), 2 to blunt force injuries and strangulation (25%), 1 to gunshot wounds and sharp force injuries (8,33%) (Fig. 5). No cases of combined injury due to three different methods were found, the case presented being the first. The victims had an average age of 54.3 years and a majority were females (7 out of 12 cases), confirming, albeit with a small sample, previous statements on the prevalence of female victims [26, 27].

## Discussion

Homicides committed using multiple methods are not uncommon in the forensic context. The case presented is a clear example in which autopsy findings, radiological, and toxicological investigations identified the sequence of injuries and corroborated the statements of the perpetrator. This case can therefore be classified as a “combined homicide” of a planned nature (as also stated by the perpetrator), since all three tools used (a hammer, a cable, and a knife) were found in a room where their placement seemed unusual, indicating premeditation. This finding further supports existing literature, which suggests that such homicides are often the result of premeditation. The case also confirms findings regarding the higher incidence of female victims and the most commonly reported methods of killing, including sharp force trauma, blunt force trauma, and strangulation, as well as the premeditation of the act [26–30].

In this case, premeditation and planning were likely motivated by a desire for “certainty” in causing the victim’s death, perhaps not directly related to the possibility of resistance from the victim herself (who was already suffering from paralysis and in need of assistance and therefore intuitively unable to offer considerable resistance, which is also partly evidenced by the absence of defensive injuries), but rather a conscious assessment by the perpetrator of his own limited strength and ability to resist. This likely led him to believe that the use of a single method would have been insufficient. Additionally, the familial relationship between the two individuals and the possible “emotional” desire on the part of the perpetrator to cause the death as quickly as possible, without prolonging the agonal phase, could be considered.

There are, therefore, similarities in terms of the different methods used, which do not belong to the same forensic classification category (blunt force, sharp force, strangulation), with cases of “complex suicide”. However, this case also differs from the concept of “overkill” because the methods used in sequence were intended to cause death, not to inflict post-mortem harm on the victim. In contrast, overkill typically refers to the excessive application of force or violence that goes beyond what is necessary to ensure death. From a medico-legal standpoint, it must be assessed that the death was caused by a combination of these different mechanisms, despite the fact that at least two of these methods (hammer blows to the head and stabbings to the abdomen) would have been sufficient on their own to ensure the victim’s death. While it is possible that the third method (strangulation with the cable) could have been sufficient on its own, this cannot be confirmed with certainty. However, it can be conclusively assessed that it significantly contributed to the victim’s death.

The use of multiple methods complicates the determination of the primary cause of death and can make forensic investigations more challenging. A detailed analysis of all the findings is essential to identify the methods of injury, as well as the sequence and/or simultaneity of the different methods and their role in determining the cause of death. It should also be noted that, while in this case there was a single perpetrator, cases of complex homicide can also involve multiple perpetrators; therefore, medico-legal analysis can also clarify “who did what”.

It is important to emphasize the need for a uniform nomenclature in such cases to provide greater clarity in forensic pathology. It is believed that the most appropriate and practical term to use is “combined homicide,” which can further be differentiated into “planned” and “unplanned” based on whether the tools were premeditatedly prepared before the crime or more casually found at the scene and adapted for the commission of the act. Although in the present case two of the three methods would have potentially been fatal on their own, it was the “combined” effect (hence the term “combined homicide”) that was responsible for the victim’s death. This is of utmost importance for a correct and unambiguous interpretation of such cases in the forensic field.

Moreover, understanding combined homicides requires a multidisciplinary approach, incorporating insights from criminology, forensic psychology, and investigative techniques. Such cases may reveal underlying patterns of behavior, the degree of premeditation and the intent to control or inflict suffering on the victim. As forensic science continues to evolve, the examination of mixed-method homicides will be crucial for developing effective investigative strategies and improving our understanding of criminal behavior.

## Data Availability

The Authors confirm that the data supporting the findings of this study are available within the article.
